# Modification of Composite Separation Membranes with Citric Acid and Metal Ion Chelation Coatings for Oil–Water Separation

**DOI:** 10.3390/polym18121450

**Published:** 2026-06-10

**Authors:** Liming Xia, Weilin Wu, Xinyi Wang, Zezhen Zhang, Haolan Xiao, Lili Wu

**Affiliations:** 1 School of Materials Science and Engineering, Wuhan University of Technology, Wuhan 430070, China; 2 School of Pharmaceutical Sciences, Hunan University of Medicine, No. 492 South Jinxi Road, Huaihua 418000, China

**Keywords:** oil–water separation, hydrophilic antifouling, recyclable

## Abstract

The development of advanced and efficient oil–water separation technologies is crucial, and membrane fouling remains one of the primary obstacles hindering the sustainable development of membrane technology. Separation membranes, which differ in pore size and material composition, can be selected based on specific environmental conditions and application requirements. In the study, a composite enhanced PVDF membrane with a complex coordination aggregation structure and abundant hydroxyl groups was prepared by introducing a citric acid–Fe(III) complex coating onto the PVDF surface using tannic acid as an interfacial adhesive layer. Citric acid (CA) molecules participate in competitive cross-linking. The carboxylic acid groups (-COOH) of CA form ionic bonds with Fe^3+^. This promotes the formation of a complex cross-linked network inside the coating. It also successfully introduces additional hydrophilic groups. Consequently, a TA-Fe/CA composite coating system is constructed. The optimized modified membrane exhibited superior performance, with a water contact angle (WCA) of 14° and complete wetting within 0.5 s. The pure water flux reached 20,473.16 L/m^2^·h. Compared to the pristine membrane, the modified membrane demonstrated significantly enhanced hydrophilicity, underwater oleophobicity, and antifouling properties. During the separation of surfactant-stabilized toluene emulsions, the PVDF-TA-Fe/CA membrane showed higher separation efficiency and permeate flux than both the PVDF and PVDF-TA membranes. Furthermore, the modified membrane demonstrated excellent chemical stability, long-term durability, and thermal stability.

## 1. Introduction

Membrane separation, as an efficient and environmentally friendly separation technology, has demonstrated broad application prospects in oil–water separation, seawater desalination, biotechnology, and chemical industries [1,2,3]. Conventional polyvinylidene fluoride (PVDF) membranes are prone to oil droplet adsorption and organic matter deposition during operation, which leads to membrane pore clogging, water flux decline, and reduction in separation efficiency. Currently, numerous researchers are striving to address the performance limitations and membrane fouling issues of PVDF membranes [4]. Xu et al. [5] fabricated ZnO/PVDF/ODA composite membranes on copper mesh via magnetron sputtering, hydrothermal, and impregnation methods. The membranes exhibited excellent mechanical and chemical stability, achieved oil–water separation efficiencies exceeding 97% for all tested mixtures, and demonstrated good cyclic stability. In recent years, tannic acid (TA)-based metal–phenolic networks (MPNs) have attracted increasing attention due to their green preparation process and strong adhesion to various substrates [6,7]. However, the application of ternary coordination complexes of tannic acid/ferric ion/citric acid (CA) for the modification of PVDF membranes used in oil–water separation has rarely been reported. In this work, we fabricated a novel tannic acid/ferric ion/citric acid composite modified PVDF membrane via a deposition method, aiming to simultaneously enhance the hydrophilicity, underwater superoleophobicity, and antifouling performance of the membrane.

With the rapid development of petrochemical, marine transportation and other industries, the discharge of oily wastewater has been increasing continuously, posing a serious threat to the ecological environment and human health [8,9]. Inspired by the unique wetting behavior of fish scales, separation membranes with superhydrophilic/underwater superoleophobic properties have attracted considerable attention from both academia and industry in recent years [10,11]. The dense and stable hydration layer formed on the membrane surface in aqueous environment can effectively prevent oil droplets from penetrating into the membrane pores and adhering to the membrane surface, thereby greatly alleviating membrane fouling [12]. This provides a practical and feasible solution for the long-term stable operation of membrane separation technology in the field of oil–water separation.

At present, the main superhydrophilic modification methods for polymer surfaces include grafting, plasma treatment, nanocoating and gel modification [13,14,15]. However, these methods generally suffer from complex synthesis, tedious preparation, use of harmful substances and difficulty in large-scale industrial production, which severely limit their practical applications. Therefore, it is of vital importance to develop superhydrophilic/underwater superoleophobic membranes with both hydrophilic chemical components and surface micro–nano-rough structures through a simple, green and environmentally friendly approach. Rather et al. [10] fabricated reactive nanocomposite coatings on polyurethane fibrous substrates via a 1,4-conjugate addition reaction between amine and acrylate groups, obtaining stretchable and durable membranes with superhydrophobicity both in air and under oil. The membranes resisted 1000 cycles of 150% stretching deformation and achieved over 99% efficiency for separating both light and heavy oil–water mixtures at a flux of approximately 115 mL·min^−1^. Chu et al. [9] systematically reviewed the design principles, fabrication, and oil–water separation applications of superhydrophobic/superoleophilic and underwater superoleophobic materials, highlighting that surface chemistry and micro/nano-roughness are essential for high-performance separation. Li et al. [8] developed superoleophobic/superhydrophilic surfaces in air by tuning the polar component of surface tension and proposed a ferroconcrete-like reinforced structure, enabling the coating to withstand abrasion over 400 m while realizing controllable oil transport and emulsion demulsification. Rather et al. [13] further extended this covalent coating strategy to natural cotton fibers, yielding superhydrophobic cotton with an oil absorption capacity above 2000 wt%. It could remove floating oil, sedimented oil, and water-in-oil emulsions by combining absorption and filtration, retaining more than 95% separation efficiency after 100 reuse cycles.

In terms of hydrophilic anti-contamination modification, researchers explored multiple strategies to improve surface performance of PVDF membranes [16]. For instance, surface grafting techniques have been employed to introduce hydrophilic functional groups—such as hydroxyl, carboxyl, or amino groups [17,18,19,20,21]—thereby significantly improving the hydrophilicity of the membranes. In particular, UV-initiated graft polymerization can be used to attach hydrophilic monomers—like acrylic acid (AA) or polyvinyl alcohol (PVA)—onto the PVDF membrane surface [22,23], forming a dense hydrophilic layer that effectively prevents the adsorption and deposition of pollutants. In addition to grafting, surface coating modification serves as another effective approach to enhance the hydrophilicity and antifouling performance of PVDF membranes [24,25,26,27,28,29,30,31,32,33,34]. Through physical adsorption or chemical bonding, hydrophilic polymers or inorganic nanomaterials—such as polyethylene glycol (PEG), graphene oxide (GO), or silica (SiO_2_)—can be applied onto the membrane surface [35,36]. These coatings not only significantly improve the wettability and fouling resistance of the membranes but also minimize direct contact between pollutants and the membrane surface. Moreover, they further inhibit the adsorption and accumulation of contaminants through steric hindrance and charge repulsion mechanisms [37,38].

The combination of tannic acid (TA) and Fe^3+^ has emerged as a novel modification method with significant potential [39]. TA is a natural polyphenol compound with rich hydroxyl functional groups that can significantly improve the material’s hydrophilicity and pollution resistance properties [40,41]. Meanwhile, Fe^3+^ forms a stable cross-linked structure through coordination with TA, further improving the stability and durability of the modified membrane [42,43]. Zhang et al. [44] reported a biomimetic coating approach in which alkali-treated PVDF membranes were functionalized with an Fe^3+^-TA-glutathione (GSH) hydrophilic coating. The optimized modified membrane exhibited a pure water flux of 3368.0 ± 94.2 L·m^−2^·h^−1^ and a toluene/water emulsion oil rejection rate of 99.3 ± 0.2%. After cyclic fouling regeneration tests simulated using bovine serum albumin (BSA) solution, the membrane achieved a high flux recovery rate of up to 97.0%. The polyphenol hydroxyl (-OH) in TA molecules are easily oxidized into quinone-like structures, accompanied by the production of strong adhesion, making them ideal materials for building multifunctional coatings [45,46,47].

The present study aimed to develop a novel composite membrane surface modification strategy based on tannic acid–iron coordination chemistry and systematically fabricate high-performance antifouling oil–water separation membrane. The specific research contents were as follows: (1) Using tannic acid (TA) as an intermediate adhesive coating and ferric ions (Fe^3+^) as a cross-linking agent, a stable TA-Fe base coating was prepared via coordination complexation. Citric acid (CA) molecules were subsequently introduced for competitive cross-linking [48]. The ionic bonding interactions between CA’s carboxylic acid groups (-COOH) and Fe^3+^ facilitated the formation of a complex cross-linked network within the coating while introducing abundant hydrophilic groups, thereby establishing a robust TA-Fe/CA composite coating modification system. (2) Key experimental parameters, including TA modification time, reaction pH, and Fe^3+^/CA molar ratio, were systematically investigated to explore their effects on the surface hydrophilicity and antifouling properties of the modified membranes and identify the optimal preparation conditions. (3) A comprehensive suite of characterizations and performance tests were conducted to evaluate the regulatory role of the TA-Fe/CA composite coating in modulating membrane hydrophilicity, antifouling performance, and long-term oil–water separation stability. This study provides valuable experimental data and theoretical insights for the rational design and fabrication of advanced antifouling oil–water separation membranes.

## 2. Materials and Methods

### 2.1. Chemicals and Materials

Polyvinylidene fluoride (PVDF) membranes (average pore size: 0.22 μm; PVDF membrane thickness: 150 μm; porosity: 77.5% ± 2.5%) were purchased from Xiamen Guochi Technology Co., Ltd. (Xiamen, China). Tannic acid (TA), ferric chloride hexahydrate (FeCl_3_·6H_2_O), citric acid (CA), Na_2_SO_3_, and K_2_S_2_O_8_ were supplied by Aladdin Reagent Co., Ltd. (Shanghai, China). Hydrochloric acid (HCl), sodium hydroxide (NaOH), Tween-80, anhydrous ethanol, and toluene were obtained from Sinopharm Chemical Reagent Co., Ltd. (Shanghai, China). Tris(hydroxymethyl)aminomethane (Tris) was provided by Baide Pharmaceutical Technology Co., Ltd. (Shanghai, China). Deionized water was produced using a deionized water generation system (RX-108, Xinrui, Shanghai, China). All chemical reagents were of analytical grade and were used without further purification.

### 2.2. Preparation of TA-Fe/CA-Modified PVDF Membranes

The PVDF microfilter film was cut to the appropriate size and soaked in deionized water for 30 min to remove water-soluble impurities and additives from the film surface. The membranes were then taken out and soaked in anhydrous ethanol for another 30 min to eliminate oil-soluble impurities, grease, and other contaminants from the membrane pores. After removal, the PVDF membranes were thoroughly rinsed with deionized water until clean. The cleaned membranes were stored in sealed containers filled with deionized water until use.

Tromethamine–chloric acid (tris-HCl) buffer solution with pH = 8.5 was used. A designated mass of TA was dissolved in the Tris-HCl buffer to prepare the TA-Tris buffer solution, which was stirred uniformly using a magnetic stirrer. The cleaned PVDF membranes were immersed in the TA-Tris buffer solution and reacted for 12 h at 25 °C under aerobic conditions.

**Figure 1:**
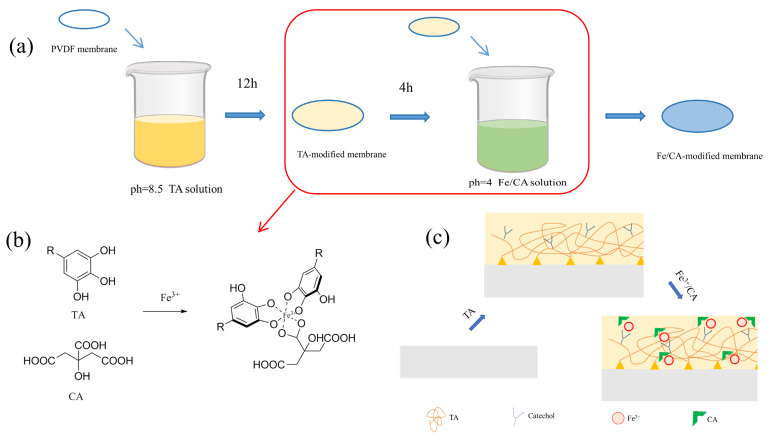
(**a**) Experimental procedure flowchart; (**b**) mechanism of Fe^3+^/TA/CA complexation; (**c**) schematic diagram of the coating formation process.

FeCl_3_·6H_2_O and CA were weighed at designated concentrations to prepare an Fe/CA solution, and the pH was adjusted accordingly. After the 12 h reaction, the PVDF membranes were taken out, rinsed with deionized water to remove residual TA solution on the surface, and then placed into the Fe/CA solution for further reaction for 4 h (Figure 1). Upon completion of the reaction, the membranes were taken out, washed with deionized water, and prepared for subsequent performance tests. The modification conditions, including TA modification time and concentrations of Fe^3+^ and CA, are shown in Table 1.

### 2.3. Surface Morphology and Elemental Analysis

The original PVDF film, TA-modified film, and Fe/CA-modified film were washed with deionized water, and then placed in a constant-temperature blast dryer to dry at 50 °C for 3 h to remove residual moisture from the film to ensure the quality and performance of the film. Subsequently, surface morphology characterization and elemental analysis tests were conducted.

The changes in the surface functional groups of the PVDF primary membrane, TA-modified membrane, and Fe/CA-modified membrane were analyzed by the Fourier Transform Infrared Spectrometer (ATR-FTIR) test, and the infrared graph was analyzed in detail. Field emission scanning electron microscopy (SEM) was employed to observe the surface pore morphology of the pristine PVDF membrane, TA-modified membrane, and Fe/CA-modified membrane at magnifications of 20,000×, 50,000×, and 100,000×. Atomic force microscopy (AFM) was utilized to examine the surface morphology and roughness. X-ray photoelectron spectroscopy (XPS) was conducted to analyze changes in elemental content on the membrane surface, and peak fitting analysis was performed on the XPS spectra of C and O elements.

### 2.4. Wettability Test

The water contact angle (WCA) and underwater oil contact angle (UOCA) of the PVDF membrane and its modified counterparts were measured using a contact angle goniometer (JC2000C, Zhongchen, China). Deionized water and dichloromethane were used as the test liquids for WCA and UOCA, respectively.

The water contact angle and underwater oil contact angle reflect the membrane’s hydrophilicity and oleoporicity, respectively. Smaller water contact angles indicate better hydrophilicity, and larger oil contact angles indicate better oleoporicity. The membranes to be tested were evenly attached to glass slides using double-sided adhesive tape for contact angle measurement. The droplet volume for both water and oil was controlled at 2 μL. Five measurements were taken at different locations on each sample, and the average value was used as the analytical result.

The pure water flux of the membrane was measured using a vacuum filtration system with an effective area of 12.56 cm^2^. The pressure was maintained at 0.5 bar, and the membrane was pre-pressurized with pure water for 3 min before testing. The flux was recorded after reaching a stable value, and the test was repeated three times to obtain an average value. The pure water flux of the membrane was calculated using Equation (1) as follows:

(1) $$J=\frac{V}{A\times t\times ∆P}$$

*J* is the pure water flux (L·m^−2^·h^−1^·bar^−1^), *V* is the volume of permeated pure water (L), *A* is the effective filtration area of the membrane (m^2^), *t* is the time required to complete the filtration (h), and Δ*P* is the transmembrane pressure difference (bar).

### 2.5. Oil–Water Separation Test

An oil-in-water emulsion was prepared using toluene. Toluene and water were mixed at a volume ratio of 1:99 to prepare 1 L of oil–water mixture. Then, 20 mg of Tween-80 was added as an emulsifier, and the mixture was stirred vigorously for 60 min to obtain a white emulsion. For the oil–water separation experiment, a vacuum filtration system was employed with a pressure set at 0.5 bar and an effective filtration area of 12.56 cm^2^. The membrane to be tested was wetted with deionized water, placed into the filtration device, and securely fixed. The emulsion was then poured into the filtration device to initiate the test, and the time required for oil–water separation was recorded. The permeate flux *J* (Equation (1)), separation efficiency *SE* (Equation (2)), and flux recovery ratio *FRR* (Equation (3)) of the separation membrane were calculated using the following formulas:

(2) $$SE\left(\%\right)=\left(1-\frac{C_{0}}{C_{1}}\right)\times 100$$

(3) $$FRR=\frac{J_{W1}}{J_{W2}}\times 100$$

The oil concentration was measured using ultraviolet–visible spectrophotometry, where *C*_0_ and *C*_1_ represent the oil concentrations in the oil-in-water emulsion and the filtrate, respectively. *J_W_*_1_ and *J_W_*_2_ denote the water flux of the membrane before and after filtering the oil-in-water emulsion, respectively.

### 2.6. Antifouling Performance Test

To explore the mechanism of interaction between the surface coating of modified membranes and oil droplets, oil adhesion experiments were conducted on the original membranes and various modified membranes. In the oil adhesion experiment, approximately 2 μL of dichloromethane was slowly extruded using a syringe needle and brought close to the membrane surface. Upon contact, the oil droplet deformed. The syringe was then gradually lifted upward, and the adhesion behavior between the oil droplet and the membrane surface was observed. To further study the antifouling properties of the membranes, the PVDF, PVDF-TA, PVDF-Fe, PVDF-CA, and PVDF-Fe/CA membranes were wetted with deionized water and immersed in toluene containing dissolved Rose Bengal B dye. After removal, the membranes were rinsed with deionized water, and the surface staining conditions were observed and recorded.

### 2.7. Stability Test

The actual working environment of oil and water insulating membranes is complex, with high requirements for the stability of the membranes. Test analysis of acid, salt, alkaline, high temperature, and long-term stability performance was performed to fully evaluate the stability of the insulating membranes in different use environments. Modified membranes were separately immersed in ethanol, 0.1 mol/L NaCl solution, 0.1 mol/L HCl solution, and 0.01 mol/L NaOH solution for 10 h continuously. Every 2 h, the membranes were taken out and rinsed with deionized water, and their water contact angles were measured to assess chemical stability. For thermal stability evaluation, the modified membranes were immersed in deionized water at 70 °C for 10 h. Similarly, they were removed and rinsed every 2 h, followed by water contact angle measurement. Long-term stability was assessed by immersing the membranes in deionized water for an extended period. During immersion, a magnetic stirrer was used to continuously agitate the water, ensuring uniform and constant flow over the membrane surface. Water contact angles and pure water flux were measured every 24 h, and the trends in their changes were analyzed.

## 3. Results

### 3.1. Modification Conditions

**Figure 2:**
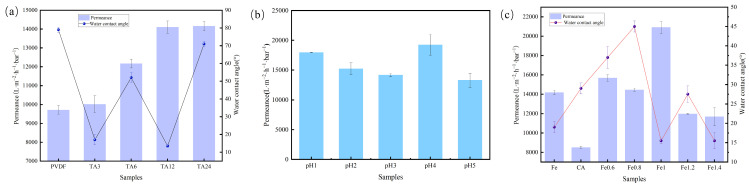
(**a**) Pure water flux and water contact angle measurements as a function of TA modification time; (**b**) pure water flux at different pH values; (**c**) pure water flux and water contact angle measurements as a function of Fe^3+^/CA molar ratio.

The pure water flux (PWF) and water contact angle (WCA) measurements of the modified membranes are presented in Figure 2a. With increasing TA modification time, the pure water flux of the membranes increased progressively. The optimal TA modification duration was determined to be 12 h, at which point the WCA further decreased to 14°, corresponding to the most hydrophilic surface achieved. A uniform and stable TA coating formed on the membrane surface, with no significant aggregation or delamination observed. For membranes modified for 24 h, the WCA rebounded to 78°, approaching that of the pristine PVDF membrane. This indicated that prolonged modification caused excessive TA deposition, forming a dense coating that hindered interactions between water molecules and polar groups on the membrane surface. Based on the combined PWF and WCA results, the membrane modified for 12 h exhibited the best overall performance (WCA = 14°, PWF = 14,271.34 L·m^−2^·h^−1^·bar^−1^). Therefore, a TA modification time of 12 h was selected for subsequent pH optimization experiments.

The TA-modified membranes were subsequently immersed in a mixed Fe^3+^/CA solution to form a Fe/CA complex coating, aimed at further enhancing the hydrophilicity and antifouling properties of the PVDF membranes. pH significantly affected the coordination stoichiometry between Fe^3+^ and CA as well as their supramolecular aggregation behavior by altering the protonation state of phenolic hydroxyl groups. Under strongly acidic conditions (pH < 3), CA molecules formed linear oligomers with Fe^3+^ via monodentate coordination. As pH increased to 4, deprotonated pyrogallol groups triggered multidentate coordination, leading to the formation of three-dimensional (3D) network-like metal–polyphenol nanoclusters. Under alkaline conditions (pH > 7), the coordination equilibrium shifted toward dissociation, resulting in a loose and unstable coating structure. As shown in Figure 2b, the membrane exhibited the best performance at a pH of 4, with a PWF of 20,473.16 L·m^−2^·h^−1^·bar^−1^. At this pH, Fe^3+^ carried a high positive charge, facilitating its binding to negatively charged or coordinatively active CA functional groups and forming a stable and dense complex network. This network structure not only optimized the pore distribution within the coating but also enhanced water permeability and reduced non-specific adsorption sites, which contributed to the high flux and excellent antifouling performance observed. Accordingly, a pH of 4 was chosen as the optimal condition for subsequent experiments.

During the mixed Fe^3+^/CA modification, the WCA showed a complex trend with varying Fe^3+^/CA molar ratios, which originated from the competitive adsorption and interactions between Fe^3+^ and CA on the membrane surface. The minimum WCA of 15° was achieved at an Fe^3+^/CA molar ratio of 1 (Figure 2c), indicating the most pronounced synergistic effect between Fe^3+^ and CA at this ratio, resulting in a surface structure most favorable for hydrophilicity. Membranes modified with Fe^3+^ alone exhibited an increased PWF of 14,331.21 L·m^−2^·h^−1^·bar^−1^, confirming that Fe^3+^ introduction effectively improved membrane permeability. In contrast, membranes modified with CA alone showed a decreased PWF of 8430.12 L·m^−2^·h^−1^·bar^−1^, which was attributed to partial pore blocking caused by the reaction between CA and membrane surface functional groups. At an optimal Fe^3+^/CA molar ratio of 1, the PWF reached the maximum value of 20,473.16 L·m^−2^·h^−1^·bar^−1^, as the synergistic effect between Fe^3+^ and CA at this ratio was most conducive to the formation of a pore structure and surface properties with excellent permeability. However, further increasing or decreasing the Fe^3+^/CA molar ratio led to a reduction in PWF, possibly due to the uneven distribution of modifiers on the membrane surface, which resulted in a membrane structure less favorable for water molecule permeation.

Overall, the modification of TA-PVDF membranes with different Fe^3+^/CA molar ratios resulted in significant changes in WCA and PWF. These changes were mainly governed by the interactions between modifiers and the membrane surface, competitive adsorption among modifiers, and the resulting coating structure. The modified membrane exhibited the best combination of PWF and WCA under the conditions of 12 h TA modification, a pH of 4, and an Fe^3+^/CA molar ratio of 1. These conditions were therefore adopted for all subsequent experiment.

### 3.2. Membrane Characterization

#### 3.2.1. Surface Morphology Analysis

**Figure 3:**
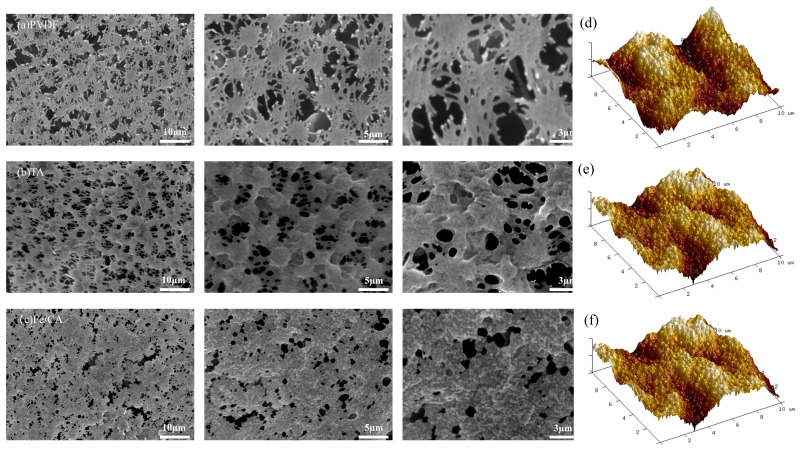
Scanning electron microscopy images of PVDF (**a**), PVDF-TA (**b**), and PVDF-TA-Fe/CA (**c**) membranes at magnifications of 20,000×, 50,000×, and 100,000×. AFM Images of PVDF (**d**), PVDF-TA (**e**), and PVDF-TA-Fe/CA (**f**) Membrane Surfaces.

The surface SEM images of the PVDF membrane, TA-modified membrane, and Fe/CA-modified membrane were analyzed (Figure 3). The pristine PVDF membrane exhibited a distinct porous structure with pores of varying sizes and shapes, indicating an uneven pore distribution. After TA modification, the surface morphology of the membrane changed significantly: the pore size decreased noticeably, and the attachment of TA molecules on the membrane surface led to smaller, more uniformly sized pores. Upon further modification with Fe/CA, the porosity of the membrane was further reduced. Compared with the pristine and TA-modified membranes, the Fe/CA-modified membrane surface showed substantial deposition of Fe/CA complexes, forming a clearly visible, large-scale coating layer. This is attributed to the rapid formation and deposition of the complexes on the membrane surface, generating bulky aggregated structures that further reduced the pore size and number of pores.

The Ra and Rq values of the pristine membrane were 71.2 nm and 55.2 nm, respectively. After TA modification, the Ra and Rq of the PVDF-TA membrane increased to 74.0 nm and 58.5 nm, showing a rise compared to those of the pristine membrane (Figure 3). This increase in roughness can be attributed to the interaction between TA molecules and PVDF chains, which induced rearrangement of the surface segments and the formation of new cross-linked structures, thereby altering the surface morphology of the membrane. For the Fe/CA-modified membrane, the Ra and Rq values further increased to 86.8 nm and 69.7 nm, respectively, representing a significant enhancement compared to both the pristine membrane and the PVDF-TA membrane. This is due to the formation of more complex composite microstructures on the surface of the Fe/CA-modified membrane, resulting in more intricate morphological features.

#### 3.2.2. Chemical Structure Analysis

**Figure 4:**
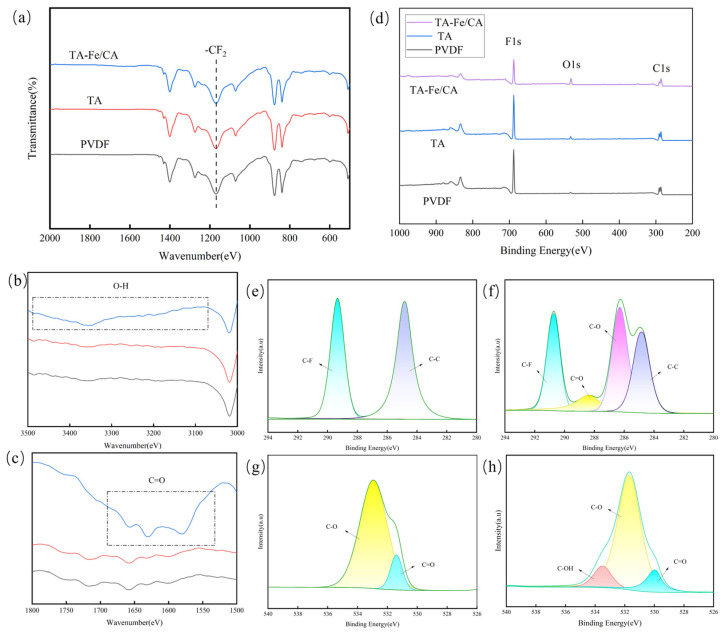
(**a–c**) Infrared spectra of PVDF, PVDF-TA, and PVDF-TA-Fe/CA membranes. (**d**) X-ray photoelectron spectroscopy of PVDF, PVDF-TA, and PVDF-TA-Fe/CA membrane surfaces; (**e**) C 1s peak fitting of PVDF; (**f**) C 1s peak fitting of PVDF-TA-Fe/CA; (**g**) O 1s peak fitting of PVDF-TA; (**h**) O 1s peak fitting of PVDF-TA-Fe/CA.

To further explore the changes in membrane surface chemical structure during modification, the infrared spectra of PVDF, TA-modified, and Fe/CA-modified membranes were analyzed using the FTIR test method. As shown in Figure 4, all membranes displayed characteristic peaks corresponding to C–C (876 cm^−1^), CF_2_ (1169 cm^−1^), and CH_2_ (1400 cm^−1^). For the TA-Fe/CA-modified membrane, a broad peak appeared in the range of 1800–1500 cm^−1^, which is associated with the stretching vibration of the carbonyl group (C=O). The emergence of this peak may be attributed to the presence of carbonyl groups in Fe/CA or to the formation of new carbonyl-containing structures between Fe/CA and PVDF, indicating successful deposition of the Fe/CA complex on the membrane surface. Furthermore, a broad peak was observed in the range of 3500–3200 cm^−1^ for the TA-Fe/CA-modified membrane, indicating the presence of hydroxyl group (O–H) stretching vibrations. This broad peak results from the hydroxyl functional groups present in the Fe/CA complex, which interact with the PVDF membrane surface through hydrogen bonding. The formation of hydrogen bonds broadens the O–H stretching vibration peak and shifts it into the 3000–3500 cm^−1^ range, providing further evidence of the successful attachment of the modified coating.

To further confirm the surface chemical composition of the samples, XPS tests were performed, and surface characteristics of PVDF, TA-modified, and Fe/CA-modified membranes were analyzed. More detailed surface information was obtained, as shown in Figure 4d. The pristine membrane contained a small amount of oxygen, which primarily originated from certain chemicals, such as pore-forming agents, inevitably introduced during the membrane fabrication process. After the first modification step, the oxygen content of the TA-modified membrane increased significantly from 2.5% in the pristine membrane to 4.08%. In the second modification step, the oxygen content of the TA-Fe/CA-modified membrane further rose to 12.65%. Concurrently, the fluorine content of the membrane gradually decreased as the modification proceeded.

In the C 1s spectra of the PVDF membrane and the Fe/CA-modified membrane (Figure 4e,f), characteristic peaks corresponding to C–F and C–C appeared at 289.3 eV and 284.8 eV, respectively. In the Fe/CA-modified membrane, additional peaks emerged at 288.35 eV and 286.29 eV, which can be assigned to C=O and C–O functional groups, respectively. TA contains abundant phenolic hydroxyl groups, which can undergo oxidation in air, converting phenolic hydroxyls to quinone groups. This process leads to a decrease in C–O content and an increase in C=O content, thereby giving rise to the observed C=O peak. Additionally, citric acid (CA) in the Fe/CA complex contains carboxyl groups, which may also contribute to the spectral features.

To obtain more accurate analytical results, the O 1s spectra of the TA-modified membrane and the Fe/CA-modified membrane were fitted (Figure 4g,h). A peak corresponding to C–OH was observed at approximately 534 eV. This peak is more likely attributed to the CA component within the Fe/CA complex. The presence of this peak indicates the successful introduction of the Fe/CA complex onto the PVDF membrane surface and its interaction with atoms on the membrane surface.

### 3.3. Wettability Analysis of the Membranes

**Figure 5:**
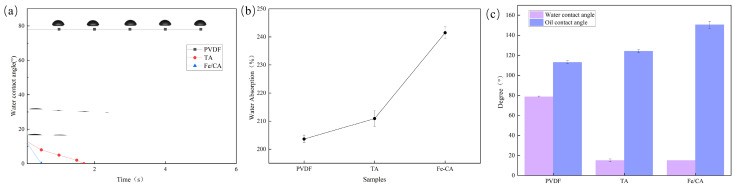
(**a**) Dynamic water contact angle; (**b**) water absorption rate; (**c**) water contact angle and underwater oil contact angle.

As shown in Figure 5a, the pristine PVDF membrane exhibited a hydrophobic interface with low surface energy due to the dense arrangement of fluorine atoms (-CF_2_-) along the molecular chains, resulting in a stable water contact angle (WCA) of approximately 78°, which resembles the water-repellent characteristics of a lotus leaf surface. The grafting of TA onto the PVDF surface via hydrogen bonding and Michael addition reactions led to a remarkable change in membrane wettability: the originally hydrophobic surface became covered with a high density of phenolic hydroxyl groups (-OH), forming a strongly polar and highly hydrophilic surface layer. This transformation significantly reduced the solid–liquid interfacial energy, causing the WCA to drop sharply to about 14°. Moreover, once a water droplet came into contact with the membrane surface, it spread rapidly and completely wetted the surface within 2 s (WCA = 0°), demonstrating typical super-hydrophilic behavior.

With the further introduction of Fe^3+^ to form a metal–phenolic network, the surface wettability of the membrane was more precisely regulated. Although the water contact angle (WCA) slightly increased to 15°—possibly due to the cross-linking effect of Fe^3+^, which made the coating more uniform and reduced excessive local clustering of hydrophilic groups—the dynamic wetting performance was significantly enhanced. Owing to the micro–nano-structure formed by the metal–phenolic composite coating, the penetration path of water molecules on the membrane surface was optimized, becoming more efficient. When a water droplet contacted the Fe/CA-modified membrane, its spreading speed was approximately four times faster than that on the TA-modified membrane, achieving complete wetting (WCA = 0°) in only 0.5 s. This demonstrates that the carboxylic acid groups (-COOH) of CA form ionic bonds with Fe^3+^. Such bonding promotes the formation of a complex cross-linked network within the coating. It also successfully introduces additional hydrophilic groups and optimizes the membrane wettability. As a result, a TA-Fe/CA composite coating system is constructed.

As shown in Figure 5b, the water absorption rate of the pristine PVDF membrane was 204%, that of the TA-modified membrane was 209%, and the further modified Fe/CA membrane exhibited a water absorption rate of 240%. All three membranes demonstrated favorable water absorption capacity, and the absorption increased progressively with the degree of modification. This trend aligns consistently with the variation in water contact angles, further confirming the correlation between the improved surface wettability and the enhanced water absorption performance of the membranes.

As shown in Figure 5c, the oil contact angle is an important indicator for evaluating the surface wettability and oleophobicity/oleophilicity of membranes. The oil contact angle of the pristine PVDF membrane was 112°, indicating a relatively strong affinity for oil droplets, which tend to spread and penetrate easily on its surface. The TA-modified membrane exhibited an oil contact angle of 123°, suggesting that the surface, enriched with hydroxyl and carboxyl functional groups, reduced its affinity for oil droplets and demonstrated a certain degree of oleophobicity. The further-modified Fe/CA membrane displayed strong oil-droplet repellency, with an oil contact angle reaching 148°, making it almost impossible for oil droplets to form or penetrate on its surface—upping to the standard for a super-oleophobic surface. This trend indicates that as the degree of modification increases, the membrane surface gradually exhibits enhanced oleophobic properties.

### 3.4. Analysis of Oil–Water Separation Performance

**Figure 6:**
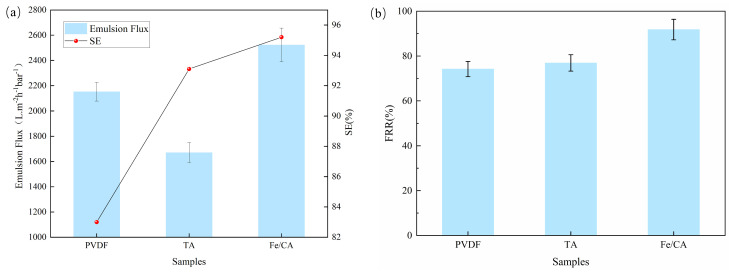
(**a**) Permeation flux and separation efficiency of the membranes; (**b**) flux recovery ratio of the membranes.

The oil–water separation performance of the modified PVDF membranes was evaluated using a toluene-in-water emulsion (stabilized with Tween-80 surfactant). The permeation flux, separation efficiency, and flux recovery rate of the pristine and modified PVDF membranes are shown in Figure 6. The pristine PVDF membrane exhibited a flux of 2204.8 L·m^−2^·h^−1^·bar^−1^ and a separation efficiency of 83% for the oil–water mixture. In comparison, the PVDF-TA membrane showed a lower permeation flux of only 1615.08 L·m^−2^·h^−1^·bar^−1^. This reduction is attributed to the dense TA coating adhered to the PVDF membrane surface, with partial deposition of TA molecules inside the membrane pores, leading to pore blockage or partial blockage. Since the toluene emulsion contains relatively large droplets, pore blockage has a more pronounced effect on flux, resulting in a noticeable decline. However, the TA modification facilitated the deposition of the Fe/CA complex, forming a more intricate and porous structure that enhanced the hydrophilicity of the membrane, thereby improving water molecule adsorption and rapid permeation. Consequently, the Fe/CA-modified membrane achieved a toluene emulsion permeation flux of 2429.03 L·m^−2^·h^−1^·bar^−1^ and a separation efficiency of 95.2%, significantly higher than those of the pristine PVDF and TA-modified membranes.

The pristine PVDF membrane exhibited a flux recovery ratio (FRR) of 74.18%, while the TA-modified membrane showed an increased FRR of 76.92%. After Fe/CA modification, the FRR was further enhanced to 91.8%. The improvement in FRR may be attributed to the combined effects of the hydrophilic layer and the protective layer formed on the modified membrane surface. TA, as a natural polyphenolic compound rich in hydroxyl functional groups, can interact with the PVDF membrane surface to form a hydrophilic layer. This layer not only enhances the hydrophilicity of the membrane but also reduces the adsorption and deposition of contaminants on the surface, thereby improving the antifouling performance of the membrane. Fe/CA modification, through its unique chemical structure and surface properties, strongly interacts with the PVDF membrane surface to form a dense protective layer. This layer effectively prevents the adsorption and deposition of pollutants and is more easily removed during cleaning, thereby improving the cleaning efficiency and flux recovery of the membrane. The hydrophilic layer facilitates the passage of water molecules through the membrane pores, while the protective layer effectively inhibits the accumulation of contaminants and reduces membrane fouling. The analysis of this synergistic effect provides new insights for membrane modification strategies.

### 3.5. Antifouling Performance Analysis

**Figure 7:**
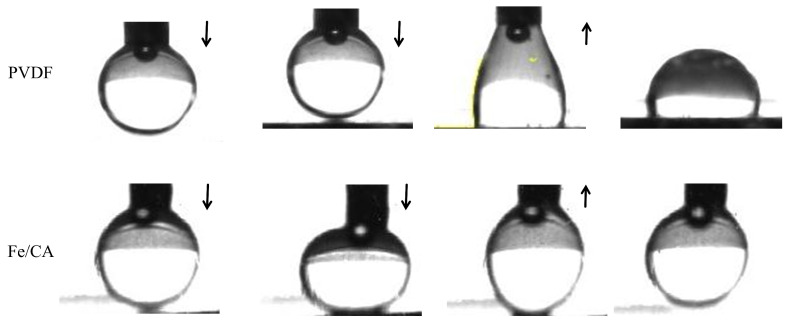
Underwater oil adhesion of the membranes.

A comparative adhesion experiment between the membrane and oil droplets was conducted to investigate the interaction between oil droplets and the membrane surface. As shown in Figure 7, approximately 2 μL of dichloromethane oil droplets was slowly moved downward toward the membrane surface. As the oil droplets gradually contacted the membrane surface, extrusion deformation was observed. The droplets were then slowly lifted upward, and their deformation behavior and adhesion status were recorded. The experimental results showed that during the slow upward movement, the droplets began to stretch and eventually detached abruptly from the syringe needle at a certain point, adhering to the membrane surface in the form of droplets. In contrast, during the adhesion experiment with the Fe/CA-modified membrane, the oil droplets did not exhibit adhesion or deformation and ultimately left the membrane surface together with the syringe needle.

The above results indicate that Fe/CA-modified PVDF membranes can significantly enhance the anti-oil adhesion properties of the original membrane, playing a crucial role in reducing the attachment of oil droplet contaminants during oil–water separation. This demonstrates that the Fe/CA-modified membrane exhibits superior antifouling capabilities, which contribute to improved stability and separation efficiency in practical applications.

**Figure 8:**
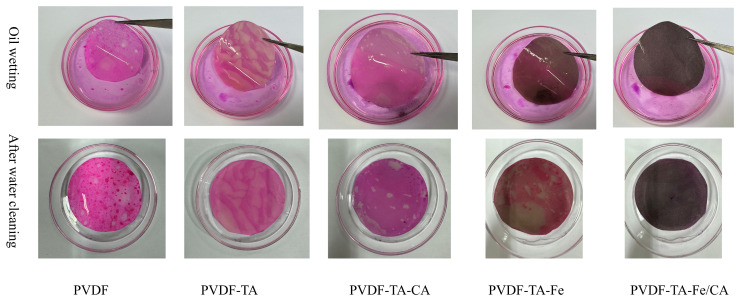
Self-cleaning test of the membranes.

To further investigate the antifouling performance, the PVDF, PVDF-TA, PVDF-TA-Fe, PVDF-TA-CA, and PVDF-TA-Fe/CA membranes were wetted with deionized water and then separately immersed in toluene containing dissolved Rose Red dye, followed by rinsing with deionized water (Figure 8). Observations revealed that the surface of the pristine PVDF membrane exhibited significant color adsorption, indicating substantial oil droplet retention, which can be attributed to the inherent low surface energy of PVDF. In contrast, the surfaces of the PVDF-TA, PVDF-TA-Fe, and PVDF-TA-CA membranes also showed color changes after rinsing, but the degree of oil contamination was markedly reduced, suggesting that TA, Fe, and CA contribute to certain antifouling capabilities.

Most notably, after rinsing with deionized water, the surface of the PVDF-TA-Fe/CA membrane remained essentially free of color deposition. This result indicates that the modified membrane maintains excellent self-cleaning performance even after exposure to high-concentration oil contamination, which is consistent with the findings from the contact angle and anti-oil adhesion experiments. The modified membrane demonstrates outstanding antifouling and self-cleaning properties, highlighting the synergistic role of TA, Fe, and CA in enhancing the membrane’s antifouling capacity. Combined with the results from Figure 8, it can be inferred that although oil droplets may temporarily reside on the surface of the PVDF-TA-Fe/CA membrane, their weak adhesion allows contaminants to be easily removed by water washing. Furthermore, the hydration layer formed on the PVDF-TA membrane surface, together with the protective barrier constructed by Fe/CA, effectively prevents direct contact between oil droplets and the membrane substrate. This dual mechanism provides additional protection to the base membrane and further improves its antifouling performance.

### 3.6. Stability Analysis

**Figure 9:**
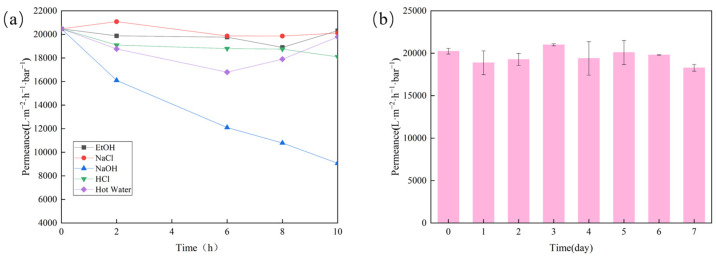
(**a**) Variation curve of pure water flux of the membranes over time under different conditions; (**b**) long-term test.

The application of modified membranes in various complex separation environments requires the coating to exhibit good stability to ensure long-term performance. Figure 9 illustrates the variation in water flux of the modified membranes over time when exposed to ethanol, NaCl (0.1 mol/L), HCl (0.1 mol/L), NaOH (0.01 mol/L), and hot water. The pristine membrane exhibited a water flux of 20,473.16 L·m^−2^·h^−1^·bar^−1^. After immersion for 10 h in ethanol, NaCl, HCl (0.1 mol/L), and hot water, the water flux changed to 20,312.12 L·m^−2^·h^−1^·bar^−1^, 20,108.23 L·m^−2^·h^−1^·bar^−1^, 18,097.87 L·m^−2^·h^−1^·bar^−1^, and 19,782.12 L·m^−2^·h^−1^·bar^−1^, respectively. The small variations indicate that the hydrophilic properties remained stable, demonstrating the favorable chemical stability of the modified membranes.

However, after 10 h of immersion in NaOH solution, the water flux decreased significantly to 9067.89 L·m^−2^·h^−1^·bar^−1^. This decline may be attributed to the reaction of Fe^3+^ with OH^−^ under strong alkaline conditions, leading to the formation of Fe(OH)_3_ precipitates. As a result, hydrogen bonds and coordination structures within the coating were disrupted, causing partial detachment or degradation of the coating and consequently reducing its hydrophilicity.

The long-term stability of the modified membranes was evaluated (Figure 9). After the membrane was immersed in water for seven days, the water flux of the modified membrane was 18,567 L·m^−2^·h^−1^·bar^−1^. Although a decrease compared to the initial flux was observed, the change was relatively small, and the flux remained at a stable level overall. This indicates that the modified membrane can maintain a favorable water flux even after prolonged immersion, demonstrating good long-term stability and the ability to retain stable separation performance in practical applications.

Overall, the modified membranes demonstrated good chemical stability in tests for acid resistance, salt resistance, and ethanol tolerance, while also exhibiting excellent thermal stability in high-temperature tests. This indicates that the modified membranes are capable of operating stably over extended periods under various harsh conditions, showing strong antifouling capabilities and promising application prospects.

## 4. Conclusions

In summary, this study successfully grafted a complex network rich in hydroxyl groups onto the PVDF membrane surface by employing tannic acid (TA) as an intermediate adhesive layer and introducing a coordination coating composed of Fe/CA and Fe^3+^. Citric acid (CA) molecules participate in competitive cross-linking. The carboxylic acid groups (-COOH) of CA presumably form coordination interactions with Fe^3+^. This promotes the formation of a complex cross-linked network inside the coating. It also successfully introduces additional hydrophilic groups. Consequently, a TA-Fe/CA composite coating system is constructed. Under the optimized conditions—a TA modification time of 12 h, Fe:CA molar ratio of 1:1, and modification solution pH of 4—the modified membrane exhibited a water contact angle (WCA) of 14° and a pure water flux of 20,473.16 L·m^−2^·h^−1^·bar^−1^. In oil–water separation tests using toluene-in-water emulsion (stabilized with Tween-80), the PVDF-TA-Fe/CA membrane demonstrated superior separation efficiency and permeation performance compared to both the pristine PVDF and PVDF-TA membranes. Future research will focus on exploring the microstructure of the coordination coating and investigating the interfacial interaction mechanisms—such as hydrogen bonding and coordination effects—between the composite coating and the PVDF substrate. Theoretical calculations could be integrated to further elucidate these mechanisms, while competitive strategies may be introduced to construct stable coatings with enhanced functionalities. Additionally, leveraging synergistic effects to improve the separation performance of membranes will be one of the key directions for future development. More importantly, considering the inherent instability of Fe^3+^ ions, future studies could rationally substitute Fe^3+^ with more stable metal ions such as Zr^4+^ and Ti^4+^ to further enhance the performance of the coating system.

## Figures and Tables

**Table 1:** Membrane modification formulas and naming.

Sample	TA Modification Time (h)	Fe^3+^ (mg/mL)	CA (mg/mL)
TA3	3	0	0
TA6	6	0	0
TA12	12	0	0
TA24	24	0	0
Fe	12	1	0
CA	12	0	1
Fe0.6	12	0.6	1
Fe0.8	12	0.8	1
Fe1	12	1	1
Fe1.2	12	1.2	1
Fe1.4	12	1.4	1

## Data Availability

The original contributions presented in this study are included in the article. Further inquiries can be directed to the corresponding authors.
